# A Modified Genetic Algorithm with Local Search Strategies and Multi-Crossover Operator for Job Shop Scheduling Problem †

**DOI:** 10.3390/s20185440

**Published:** 2020-09-22

**Authors:** Monique Simplicio Viana, Orides Morandin Junior, Rodrigo Colnago Contreras

**Affiliations:** 1 Department of Computing, Federal University of São Carlos, São Carlos, SP 13565-905, Brazil; monique.viana@ufscar.br; 2 Department of Applied Mathematics and Statistics, University of São Paulo, São Carlos, SP 13566-590, Brazil; contreras@usp.br

**Keywords:** genetic algorithm, local search, multi-crossover, job shop scheduling problem, combinatorial optimization

## Abstract

It is not uncommon for today’s problems to fall within the scope of the well-known class of NP-Hard problems. These problems generally do not have an analytical solution, and it is necessary to use meta-heuristics to solve them. The Job Shop Scheduling Problem (JSSP) is one of these problems, and for its solution, techniques based on Genetic Algorithm (GA) form the most common approach used in the literature. However, GAs are easily compromised by premature convergence and can be trapped in a local optima. To address these issues, researchers have been developing new methodologies based on local search schemes and improvements to standard mutation and crossover operators. In this work, we propose a new GA within this line of research. In detail, we generalize the concept of a massive local search operator; we improved the use of a local search strategy in the traditional mutation operator; and we developed a new multi-crossover operator. In this way, all operators of the proposed algorithm have local search functionality beyond their original inspirations and characteristics. Our method is evaluated in three different case studies, comprising 58 instances of literature, which prove the effectiveness of our approach compared to traditional JSSP solution methods.

## 1. Introduction

Scheduling problems have been extensively researched in recent years because it is a high complexity combinatorial optimization problem and it is classified as NP-Hard. Among the machine scheduling problems, there are several variations, such as Job Shop Scheduling Problem (JSSP), Flexible Job Shop Scheduling Problem (FJSSP), Flow Shop Scheduling Problems (FSP), and so forth. In this paper, we will consider the JSSP variant. JSSP is a combinatorial optimization problem composed of a set of Jobs to be processed in a set of Machines. So that to solve the problem it is necessary to find a sequence of Jobs for each Machine to optimize a specific performance criterion, for example, the makespan, which corresponds to the total processing time of all Jobs [1,2].

In recent years, several meta-heuristics approaches have been proposed to treat the JSSP, such as Greedy Randomized Adaptive Search Procedure (GRASP) [3], Local Search Genetic Algorithm (LSGA) [4], Parallel Agent-based Genetic Algorithm (PaGA) [5], Agent-based Local Search Genetic Algorithm (aLSGA) [6], Golden Ball Algorithm (GB) [7], Initial Population Based Genetic Algorithm (IPB-GA) [8], Memetic Algorithm (MA) [9], Improved Biogeography-Based Optimization (IBBO) [10], Grey Wolf Optimization (GWO) [11], Hybrid Grey Wolf Optimization (HGWO) [12], Memetic Chicken Swarm Optimization (MeCSO) [13], Genetic Algorithm with a critical-path-guided Giffler and Thompson crossover operator (GA-CPG-GT) [14] and the Discrete Wolf Pack Algorithm (DWPA) [15].

Most state-of-the-art studies on JSSP have validated the methods proposed in traditional benchmark sets in the literature, such as Fisher and Thompson [16], Lawrence [17], Applegate and Cook [18] and Adams, Balas and Zawack [19], and considered the minimization of makespan as a performance criterion for the evaluation of possible solutions of a JSSP.

We observe from the literature review that JSSP is a relevant academic topic and it has attracted the attention of many researchers because it has a combinatorial behavior and it is classified as NP-Hard, which makes it very difficult to solve by exact approaches, which encourages treatment by alternative methods such as meta-heuristics.

Several approaches of the meta-heuristic GA have been successfully performed on many combinatorial optimization problems, such as machine scheduling problems, for example, JSSP. However, for problems with greater complexity, GA needs to engage with particular problem methods to make the approach effective. Hybridization is a satisfactory and widely used way to improve the performance of GA. Local search techniques are very common forms of hybridization which have been used in several studies to improve GA performance, such as in References [4,6,20,21].

In this paper, a new GA approach with improved local search techniques (mXLSGA) is proposed to minimize the makespan in JSSP. Three local search operators are proposed, one of which is embedded in a multi-crossover operator; one as a mutation operator; and another of massive behavior. Such procedures are enhancements based on References’ [4,6,22] methods.

This paper is an extended version of our preliminary work [1]. In this manuscript, we add a section with a literature review, we also present more details in the description of the method and we conduct a deeper analysis of the experiments with more works compared and more case studies. Also, all steps of the method are outlined and detailed in the form of algorithms that simplify the reproducibility of the technique.

The sections of this article are organized as follows—in Section 2, we discussed some works that served as inspirations for the development of our technique. In Section 3, we describe the formulation of JSSP. Section 4 presents the details of the proposed mXLSGA method. In Section 5, some experimental results in the form of three different case studies are presented in order to validate our technique. Section 6 presents the conclusion and future works.

## 2. Related Works

An extensive technical background based on meta-heuristics has been presented in the literature in recent years, becoming from the most diverse inspirations, such as the well-known evolutionary algorithms of genetic inspiration [23], the behavior of students in the classroom [24], the behavior of different species of animals [25], and some theoretic-mathematical concepts like the golden ratio [26]. In particular, some of these techniques have been used successfully in the JSSP solution. In summary, to show the relevance of the problem addressed and how the most varied approaches have been proposed in recent years, we highlight some of the most recent works that use meta-heuristics to solve the JSSP:

- **Genetic Algorithm:** [4,5,6,8,20,22,27,28,29,30,31,32,33,34,35,36,37,38,39,40,41,42];
- **Ant Colony Optimization:** [43,44,45,46];
- **Particle Swarm Optimization:** [47,48,49,50,51];
- **Simulated Annealing:** [52,53,54,55];
- **Tabu Search:** [44,56,57,58];
- **Grey Wolf Optimization:** [11,12];
- **Chicken Swarm Optimization:** [13];
- **Golden Ball Algorithm:** [7];
- **Artificial Bee Colony:** [59,60];
- **Single Seekers Society:** [61];
- Among others.

We can see in the brief list above that some meta-heuristics have been more widespread in the literature in relation to the application in JSSP than others and, therefore, these meta-heuristics have greater amounts of publications in this field. For example, we can highlight GA, which was widely used in a much larger number of published works with applications in JSSP than the Single Seekers Society method, which, to the best of our knowledge, was applied to only one work in this field. This is because, among the variety of meta-heuristics that address the JSSP and present good results, GA achieved great prominence for offering a good performance due to its global research capacity. In addition, algorithms that use hybrid meta-heuristics are standing out among the various methods used in JSSP since they can combine the merits of different meta-heuristic algorithms. Hybrid algorithms generally perform remarkably well, incorporating local research to obtain an appropriate combination of diversification and intensification efforts. Among the great diversity of studies, it is possible to verify the superiority of the meta-heuristics of global search behavior with the inclusion of techniques specialized in performing a local search. This type of procedure is addressed in some works, recent or not, such as the approaches in References [4,6,9,11,12,13,22], among others.

In the following paragraphs, some of the literature that deals with the JSSP through the approach of several meta-heuristics will be detailed. The following detailed works are References [4,6,22]. We chose these techniques because they represent significant advances in the solution of the JSSP using local search strategies in GAs.

Ombuki and Ventresca [4] proposed the LSGA meta-heuristic to treat the JSSP to minimize the makespan, that is, the maximum completion time. The proposed LSGA is a GA with a local search, which has an operator similar to the mutation that is aimed at local research to further improve the quality of the solution. In LSGA, local search is applied probabilistically, that is, it applies the simple mutation or the local search mutation selected dynamically in each generation of GA. In a simple mutation, exactly one exchange is allowed, that is, the positions of two consecutive jobs selected at random are exchanged. In a local search mutation, a systematic approach is used to consecutively test multiple swaps in terms of chromosome lengths, the best improvement obtained by the swap is saved, but if no improvement was found, no swap will be saved in the original solution and the process will be finished. LSGA found solutions whose makespan results were equal to the best-known values or when worst, were within the maximum error range of 10%. LSGA obtained a better search behavior and achieved better makespan results than a canonical GA, so it is possible to say that the local search strategy included in GA improved its search technique and, with that, its solutions obtained.

In the article by Watanabe, Ida and Gen [22], the meta-heuristics GA with search area adaptation (GSA) was proposed to solve the JSSP. The proposed GSA has an adaptation of the search area with the ability to adapt to the structure of the solution space and to control the balance between global and local searches. The crossover operation of the GSA consists of performing the crossover several times on all pairs of parents, each time a new cutoff point is determined. The crossover is repeated until a better child is found than the worst individual in the population or until a certain number of iterations is reached. The mutation operation consists of executing disturbances several times on all children, performing several swaps. The mutation is repeated until a better mutant child is found than the worst individual in the population or until a certain number of iterations is reached. The GSA was compared with a GA and showed better results. The GSA achieved greater frequency in finding solutions closer to optimal solutions. Although the GSA did not find the best-known solution in the tested instances, it showed an improvement in canonical GA.

The meta-heuristic aLSGA, proposed by Asadzadeh [6], was created specifically to solve the JSSP. The proposed aLSGA is a GA with a local search with the inclusion of intelligent agents. The method consists of a multi-agent system, in which each agent has a specialized behavior to implement the local search. The aLSGA combines local search heuristics with crossover and mutation operators. The proposed method has two local search procedures, the first is called the Local Search Agent (LSA) and the second is the Elite Local Search Agent (ELSA). The first is responsible for exploring the neighborhood of each chromosome produced by the crossover operator and the second is responsible for improving the quality of the best chromosome in the population. The computational results showed that the proposed aLSGA is effective in finding optimal and almost optimal solutions for several instances of JSSP. It is noted that by including a local search heuristic in GA, an improvement is obtained in its performance and in its convergence speed.

Several approaches applied in JSSP have been proposed, some have included intelligent agents, parallel populations, or hybridization of meta-heuristics with local search techniques. It is verified through the reported works that hybridization is an effective way to improve the performance of several meta-heuristics, and presents relevant results in the literature. Local search techniques are the most common form of hybridization and are used to improve the performance of these algorithms. It is precisely in this scope that we intend to act, with the proviso that our methodology is presented as an extension and improvement of References [4,6,22] to define a GA portfolio with specialized local search for JSSP.

## 3. Formulation of Job Shop Scheduling Problem

JSSP is a combinatorial optimization problem belonging to the NP-Hard class of computational problems, which means it is a problem whose processing time is non-polynomial. Specifically, a JSSP can be defined as being a set of *N* jobs to be processed into a set of *M* machines. In JSSP, each job must be processed by all *M* machines and each job has a sequence of operations with *M* distinct elements belonging to the set of all possible operations. The sequences of operations are usually different for each job. The scheduling problem of JSSP-type production is finding a job sequence for each machine to optimize a specific performance criterion, which is usually makespan. Some restrictions must be followed in this problem [2]:

- Each job can be processed on a single machine at a time;
- Each machine can process only one job at a time;
- Operations are considered non-preemptive, that is, cannot be interrupted,
- Configuration times are included in processing times and are independent of sequencing decisions.

In this work, makespan is adopted as a measure of performance, which is considered to be the total time required to complete the production of a series of jobs. The makespan performance measurement formulation is generally used in JSSP approaches as an objective function that guides algorithms using meta-heuristics to search for appropriate solutions.

Mathematically, suppose the following specifications of JSSP:

- $J=\{J_{1},J_{2},\ldots ,J_{N}\}$ as the set of jobs;
- $M=\{m_{1},m_{2},\ldots ,m_{M}\}$ as the set of machines;
- $O=(O_{1},O_{2},\ldots ,O_{N\cdot M})$ is the sequence that defines the priority with which each job has its processing started on each of the machines of its respective script,
- $T_{i}\left(O\right)$ representing the time the job $J_{i}$ takes to be processed by all machines in its script, and thus it is considered finished according to the sequence of operations defined in *O*.

Thus Reference [62], the makespan measure of an operation sequence *O* can be defined as the value presented in Equation (1).

(1) $$\mathrm{makespan}=\max_{i}T_{i}\left(O\right),$$

which is a measure given according to the order of operations defined in *O*, since the time that each job takes to be considered finished is given according to the processing order defined in the schedule.

## 4. Multi-Crossover Local Search Genetic Algorithm for JSSP

In this section, we will discuss fundamental concepts to the execution of the proposed algorithm and we will also specify the improved methods defined in this work for better efficiency. In short, our contributions are comprised in the following topics:

- An improved crossover operator (Section 4.4) based on the version of Reference [22], including a multi-crossover strategy with the goal of increasing the search capability of the method by using a framework based on a set of crossover functions.
- An improved local search technique (Section 4.5) in union with a generalized version of the mutation operator proposed in Reference [6] and in Reference [4], including a variable parameterization.
- An improved version of the elite local search operator (Section 4.6) of Reference [6], expanding the search space by utilizing a set of mutation functions.

In this way, our contribution starts in fact from Section 4.4, with the previous Section 4.1, Section 4.2 and Section 4.3 of a descriptive and informative nature that present basic concepts of the operation of our method and most of the GA-like methods specialized in solving the JSSP.

### 4.1. Genetic Representation

Except for the presence of specific operators of each work, the basic structure of a GA continues to be formed by the repetition loop that involves two main operators: crossover operator and mutation operator. This structure is preserved in the vast majority of state-of-the-art techniques. The codification used to represent a possible solution (chromosome) of the problem can be done in many different ways, as highlighted in Reference [63].

In this paper, a codification equivalent to one of the most common representations of the literature is used, which is known as “coding by operation order”, first presented in Reference [64]. Since, in this representation, the solution space of a JSSP of *N* jobs and *M* machines is formed by chromosomes $c\in N^{N\cdot M}$, such that exactly *M* coordinates of *c* are equal to *i* (representing the job index *i*), for every i∈{1,2,…,N}.

**Figure 1:**
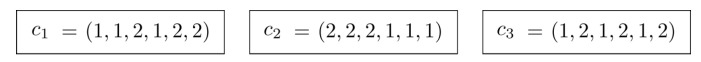
Examples of chromosomes in representation by operation order.

Figure 1 shows some examples of chromosomes $(c_{1},c_{2}$ and $c_{3})$ that obey such formulation in a JSSP with 2 jobs (N=2) and 3 machines (M=3). As the formulation requires, index job 1 and index job 2 appear exactly 3 times, since 3 is the number of machines in the problem.

This codification determines that the priority of each operation on machine allocation. As an example, let c=(1,2,1,1,2,2) be a chromosome in a 2×3 dimension JSSP. In this case, the order established by *c* defines that the following actions must be performed sequentially and it should only be initiated if it can be performed in parallel with the previous action or if the previous action has already been completed:

- (1st) Job 1 must be processed by the 1st machine of its script.
- (2nd) Job 2 must be processed by the 1st machine of its script.
- (3rd) Job 1 must be processed by the 2nd machine of its script.
- (4th) Job 1 must be processed by the 3rd machine of its script.
- (5th) Job 2 must be processed by the 2nd machine of its script.
- (6th) Job 2 must be processed by the 3rd machine of its script.

Thus, one way to generate initial population in this type of configuration is to create a group of chromosomes equal to (1,…,1,2,…,2,…,N,…,N), in which each of the *N* jobs of a JSSP appears in exactly *M* positions, and then randomly rearrange all coordinates of each chromosome. Thus, one way to generate initial population in this type of configuration is to randomly rearrange to the coordinates of each chromosome *M* representations of each of the *N* jobs of a JSSP. Mathematically, we consider the function $f_{\mathrm{shuffle}}$ that randomly reorders the coordinates of a given vector, defined in Equation (2):

(2) $$\begin{array}{cccc} f_{\mathrm{shuffle}}: & {\left\{1,2,\ldots ,N\right\}}^{N\cdot M} & ⟶ & {\left\{1,2,\ldots ,N\right\}}^{N\cdot M}\\ \; & \left(x_{1},x_{2},\ldots ,x_{N\cdot M}\right) & ⟼ & (x_{i_{1}},x_{i_{2}},\ldots ,x_{i_{N\cdot M}}), \end{array}$$

where $i_{1},i_{2},\ldots ,i_{N\cdot M}$ are a random arrangement of indices 1,2,…,N·M.

### 4.2. Fitness Function

The objective function, or fitness function, of the optimization problem discussed here can be modeled according to the function *F*, defined in Equation (3) given below:

(3) $$\begin{array}{cccc} F: & O & ⟶ & R\\ \; & O & ⟼ & F\left(O\right):=\max_{i}T_{i}\left(O\right), \end{array}$$

where O is the set of all possible sequences for the defined JSSP. That is, if O∈O, then *O* is a possible sequence of operations that defines the start processing priority for *N* jobs on *M* machines. In other words, O is the feasible set of solutions in which our method must perform its search.

The lower the makespan value of a schedule, the less time must be taken to finish a given set of jobs. Thus, the algorithm should look for configuration options for a schedule in order to minimize the time spent to complete the jobs processing on the set of machines that configure the JSSP.

### 4.3. Selection Mechanism

Selection strategies are used so that we can choose individuals to reproduce and to create new populations in evolutionary algorithms. In this paper, individuals should be selected to participate in the crossover process with probability equivalent to their fitness value, which is known as roulette wheel selection [65]. In this case, the individuals selected for crossover must define a set entitled *P*_selected_. The selection approach for generating a new population used in the proposed algorithm was the roulette wheel selection with retaining model, in which the probability of an individual being selected is proportional to its fitness value and, certainly, the best individual in the current population is transferred to the new population. It is important mentioning that there are different mechanisms for selecting individuals available in the specialized literature, however, we will focus our analysis on the models mentioned, since these models are widely used in studies that address the JSSP and present good results in this field, as is the case of the advances brought by Ombuki and Ventresca [4], Asadzadeh and Zamanifar [5] and Asadzadeh [6], which only use roulette wheel selection strategies in their work.

### 4.4. Proposed Multi-Crossover Operator

To detail the operation of our multi-crossover operator, we need to address the use of different crossover functions. In Section 4.4.1, we model the possible functions for this operator and exemplify the operation of two of the most used functions to solve the JSSP. In Section 4.4.2, we present our multi-crossover strategy that makes up our method and consists of one of the contributions of this work.

#### 4.4.1. Crossover Functions

The proposed crossover operator consists of using more than one crossover function in search area adaptation [22] strategies. Thus, our proposal is in the form of a framework that considers a set of $n_{\times }$ crossover functions to combine chromosomes. Thus, we define this set to be $F_{\times }$, presented in Equation (4).

(4) $$F_{\times }=\left\{f_{\times ,1},f_{\times ,2},\ldots ,f_{\times ,n_{\times }}\right\}.$$

Let us consider for our modeling, without loss of generality, that each function $f_{\times }\in F_{\times }$ is a function that combines two parent chromosomes from the feasible set resulting in two children chromosomes. That is, each function of $F_{\times }$ takes the form of $f_{\times }:O\times O\to O\times O$.

In this work, we will focus on two main crossover functions for conducting our assessments and evaluations: Partially Mapped Crossover (PMX) [66] and Order-based Crossover (OX2) [67]. These functions are two of the most widely used crossover functions in the specialized literature on JSSP solution by genetic algorithm. In this way, $F_{\times }=\left\{\mathrm{PMX},\mathrm{OX}2\right\}$ for our experiments, however, the same conclusions of our method can be obtained with any set $F_{\times }$.

**Figure 2:**
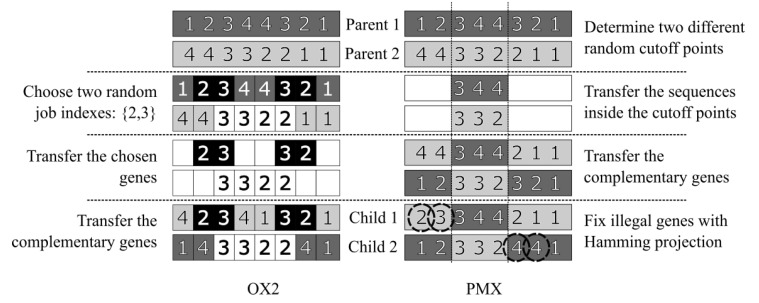
Comparison between the steps of the Order-based Crossover (OX2), on the left, and Partially Mapped Crossover (PMX), on the right, crossover techniques.

OX2 (Figure 2-left) does not require any correction or projection steps as its feasibility is guaranteed by construction. This is because the technique only matches the order in which jobs appear in parents. In detail, initially, a random number of genes are fixed. An offspring inherits in its coordinates the exact position these genes assume in one parent and completes the remaining positions with the other parent’s unfixed genes.

PMX (Figure 2-right) combines two chromosomes from two previously randomly defined cutoff points. To generate the child chromosomes, the strategy is to mix the genes internal to the cutoffs in one parent with the genes external to the cutoffs in another parent. This procedure can generate infeasible solutions, which are usually corrected [6] in JSSP applications by projecting the individuals generated into feasible space with respect to the Hamming distance [68].

To exemplify the working of these crossover functions, let’s assume that we are going to apply both functions OX2 and PMX to the same pair of individuals $Parent_{1}=\left(1,2,3,4,4,3,2,1\right)$ and $Parent_{2}=\left(4,4,3,3,2,2,1,1\right)$. In details:

- **In the case of the crossover function OX2**:
  1 Initially, it is necessary to decide what will be the values of the genes (jobs) that should maintain their positions in the coordinates of the offsprings. Let’s assume that the jobs 2 and 3 were randomly set. In this way, the genes that represent the 2 and 3 index jobs must occupy the same position in the coordinates of the offsprings generated during the transfer of the chosen genes.
  2 Therefore, the two intermediate offsprings $Child_{1}=\left(--,2,3,--,--,3,2,--\right)$, which inherits the positions of the 2 and 3 genes from $Parent_{1}$ in their coordinates, and $Child_{2}=\left(--,--,3,3,2,2,--,--\right)$, which inherits the positions of the 2 and 3 genes from $Parent_{2}$ in their coordinates. The symbol “−−” means that the coordinate of the offspring is empty.
  3 Then, it is necessary to transfer the other jobs (1 and 4) in the coordinates of the offsprings that did not receive any jobs. However, now $Parent_{1}$ will pass its genetic information to $Child_{2}$ and $Parent_{2}$ will pass its genetic information to $Child_{1}$. Then, respecting the order in which the coordinates that represent the jobs 1 and 4 in parents are arranged, the positions of these genes in the parents are transferred to the offsprings. As a result, we get $Child_{1}=\left(4,2,3,4,1,3,2,1\right)$ and $Child_{2}=\left(1,4,3,3,2,2,4,1\right)$.
  4 This crossover is completed without the need to perform any correction procedure to make the children generated feasible, since the offsprings will always be rearrangements, or shuffling, of the information contained in the parents.
- **In the case of the crossover function PMX**:
  1 This crossover takes into account just the parents’ geometric information. Thus, it is necessary to define two cutoff points randomly. Let’s assume that these cutoff points define the jobs represented by the coordinates 3, 4 and 5 as internal sequence. Consequently, the outside of these parents will be defined by the coordinates 1, 2, 6, 7 and 8.
  2 Immediately, the information contained in the parents’ internal part is passed to the children, so that the internal part of $Parent_{1}$ is passed to the internal part of $Child_{1}$ and the internal part of $Parent_{2}$ is passed to the internal part of $Child_{2}$. Thus, intermediate offsprings are defined as $Child_{1}=\left(--,--,3,4,4,--,--,--\right)$ and $Child_{2}=\left(--,--,3,3,2,--,--,--\right)$.
  3 To ensure that both parents pass genetic information to both children, in this step $Parent_{2}$ transfers its external part to $Child_{1}$ and $Parent_{1}$ transfers its external part for $Child_{1}$. Thus, we have $Child_{1}=\left(4,4,3,4,4,2,1,1\right)$ and $Child_{2}=\left(1,2,3,3,2,3,2,1\right)$.
  4 As this crossover function takes only the geometric information from the parents, it is possible that some offspring is not a feasible solution for the JSSP. And, in fact, this is what happens in this example, since $Child_{1}$ represents four times the index job 4, which would correct to represent only twice. The same occurs for $Child_{2}$ which represents index jobs 2 and 3 three times. Therefore, it may be necessary to use some techniques for projecting solutions on the feasible space of solutions. This projection is usually done according to the Hamming distance and this is how we are going to proceed in this work. Therefore, the children generated by this crossover are: $Child_{1}=\left(2,3,3,4,4,2,1,1\right)$ and $Child_{2}=\left(1,2,3,3,2,4,4,1\right)$.

A schematic of the example described is shown in Figure 2.

#### 4.4.2. Multi-Crossover Operator

As a crossover operator, a more embracing and rigorous version of the crossover operator of Reference [22] is proposed. We suppose that the use of distinct crossover techniques increases the power of local exploitation since they define different strategies to combine the same individuals and thus it allows the investigation of different search areas. Thus, the crossover operator works from three randomly selected individuals in the population and occasionally different crossover techniques defined by $F_{\times }$ is applied in all possible pairs of these three chromosomes until three offsprings are found that surpass their respective parents or until each pair has performed $R_{c}$ crossovers. Detailed operator schematics are presented in Algorithm 1.

**Algorithm 1** Proposed multi-crossover operator.
**Input:**
(*p*1, *p*2, *p*3)3 randomly selected individuals taken from *P*selected
*F*
Fitness function$F_{\times }$ = {$f_{\times }$_,1_,…,$f_{\times }$_,$n_{\times }$_}Set of crossover functions
*Rc*
Max crossovers for each pair1: **for**
*k* := 1 to 3 **do**
▷ Evaluate all possible pairs, or couples, among the three parents
2:       **if**
*k* == 1 **then**3:            (*P*_1_, *P*_2_) := (*p*_1_, *p*_2_)
▷ In the first iteration, evaluate the pair formed by *p*1 and *p*2
4:       **else if**
*k* == 2 **then**5:            (*P*_1_, *P*_2_) := (*p*_1_, *p*_3_) 
▷ In the second iteration, evaluate the pair formed by *p*1 and *p*3
6:       **else if**
*k* == 3 **then**7:            (*P*_1_, *P*_2_) := (*p*_2_, *p*_3_)
▷ In the third iteration, evaluate the pair formed by *p*2 and *p*3
8:       **end if**9:       *F*_*P*_1_ :_= *F*(*P*_1_)
▷ Evaluate the fitness of the couple selected for the *k*-th iteration
10:     *F*_*P*_2_ :_= *F*(*P*_2_)11:       **for**
*i* := 1 to *Rc*
**do**
▷ Try to get a child better than parents a maximum of *Rc* times
12:            $f_{\times }$:= pick_randomly ( {$f_{\times }$_,1_,…,$f_{\times }$_,$n_{\times }$_})
▷ Pick randomly a crossover function, where
      pick_randomly (*Y*) is a function that returns randomly some element from the set *Y*13:            (${\hat{c}}_{i,1}$, ${\hat{c}}_{i,2}$) = $f_{\times }$(*P*_1_, *P*_2_)
▷ Generate a pair of children using the selected $f_{\times }$

14:            *F_i_*_,1_ := *F*(${\hat{c}}_{i,1}$)
▷ Evaluate the first child
15:            *F_i_*_,2_ := *F*(${\hat{c}}_{i,2}$)
▷ Evaluate the second child
16:            **if**
*F_i_*_,1_ < *F_i_*_,2_
**then**
▷ What is the best offspring?
17:                        *F_i_* := *F_i_*,_1_
▷ If the first child is the best, then it should be considered for comparison
18:                        ${\hat{c}}_{i}$ := ${\hat{c}}_{i,1}$19:            **else**20:                        *F_i_* := *F_i_*_,2_
▷ If the second child is the best, then it should be considered for comparison
21:                        ${\hat{c}}_{i}$ := ${\hat{c}}_{i,2}$22:            **end if**
23:            **if**
*F_i_* < *F*_*p*_1__ or *F_i_* < *F*_*p*_2__
**then**

▷ If the best generated child is better than one of the parents
24:                        break
▷ Stop generating offsprings and get out of this loop
25:            **end if**26:       **end for**27:       i^*^ := arg $\min_{i}${*F_i_*}
▷ Take the generated child with the best fitness value
28:       *c_k_* := ${\hat{c}}_{i}$∗29: **end for****Output:** (*c*_1_, *c*_2_, *c*_3_) Generated individuals

Thus, in all possible pairs of three individuals randomly taken from a set of pre-selected individuals *P*_selected_, a set of distinct crossover functions are eventually performed until a solution is generated that has a fitness value better than a parent’s fitness value or until the algorithm performs $R_{c}$ crossover.

The search criteria of this operator is far stricter than the search criteria of the operators of Reference [22], since the operators of these authors perform crossover until a solution is found whose fitness value is better than the worst fitness value presented in the entire population, and the fitness value of parents is not necessarily important for the procedure. Therefore, the proposed operator should be able to find good solutions more easily than the crossover operator of Reference [22], since it performs a more careful and strict search. Furthermore, the use of different crossover techniques increases the search on feasible space, since the solutions must be generated by distinct crossover methodologies and, therefore, the search area can be explored by distinct strategies.

### 4.5. Proposed Mutation Operator

To detail our mutation operator, we need to discuss the use of different mutation functions. In this way, in Section 4.5.1, we model the possible functions for this operator, and we exemplify the application of three functions widely used in JSSP. In Section 4.5.2, we present the strategy of a local search associated with the mutation operator that makes up our method and consists of one of the contributions of our work.

#### 4.5.1. Mutation Functions

Similar to the crossover operator, the proposed mutation operator works according to a set of $n_{mut}$ mutation functions in a framework. That is, in the mutation process performed in the proposed method, a chromosome may be mutated with a mutation specified by one of the functions of set $F_{mut}$, defined in Equation (5).

(5) $$F_{\mathrm{mut}}=\left\{f_{\mathrm{mut},1},f_{\mathrm{mut},2},\ldots ,f_{\mathrm{mut},n_{\mathrm{mut}}}\right\},$$

where each function $f_{\mathrm{mut}}\in F_{\mathrm{mut}}$ is a mutation function that operates with respect to two coordinates *i* and *j* of a chromosome, making these functions of the form presented in Equation (6).

(6) $$\begin{array}{cccc} f: & O\times {\left\{1,2,\ldots ,N\cdot M\right\}}^{2} & ⟶ & O\\ \; & \left(c,(i,j)\right) & ⟼ & f(c,(i,j)) \end{array}.$$

In our work, we focus on the three most commonly used mutation functions in machine scheduling problems: Swap, Inverse and Insert [69], respectively represented by the functions $f_{\mathrm{swap}}$, $f_{\mathrm{inverse}}$ and $f_{\mathrm{insert}}$. Thus, all tests performed on our evaluations are made according to $F_{\mathrm{mut}}=\{f_{\mathrm{swap}},f_{\mathrm{inverse}},f_{\mathrm{insert}}$, the same considerations are made generally.

We will represent the operation of these mutation functions with an example. Suppose we are going to perform perturbations using these three functions on chromosome c=(4,3,2,3,2,4,1,1) and considering the same pair of coordinates (i,j)=(3,8). Therefore, the mutation functions must perturb *c* as follows:

- **$f_{\mathrm{swap}}\left((4,3,2,3,2,4,1,1),(3,8)\right)$**: The job represented by the coordinate i=3, which in this case is the index job 2, must change places with the job represented by the coordinate j=8, which is the index job 1. Thus, the mutant form of *c* by the $f_{\mathrm{swap}}$ function is (4,3,1,3,2,4,1,2).
- **$f_{\mathrm{inverse}}\left((4,3,2,3,2,4,1,1),(3,8)\right)$**: All coordinates between i=3 and j=8 are inverted, acting as an extension of the $f_{\mathrm{swap}}$ function that also changes the internal values between the coordinates 3 and 8. Therefore, the mutant form of *c* by the $f_{\mathrm{inverse}}$ function is (4,3,1,1,4,2,3,2).
- **$f_{\mathrm{insert}}\left((4,3,2,3,2,4,1,1),(3,8)\right)$**: The job represented by the coordinate j=8 is inserted in the coordinate subsequent to the coordinate i=3, that is, in the fourth coordinate. Then, jobs represented by the coordinates between i=3 and j=8 are transferred to a position ahead. Thus, the mutant chromosome generated by $f_{\mathrm{insert}}$ is (4,3,2,1,3,2,4,1).

**Figure 3:**
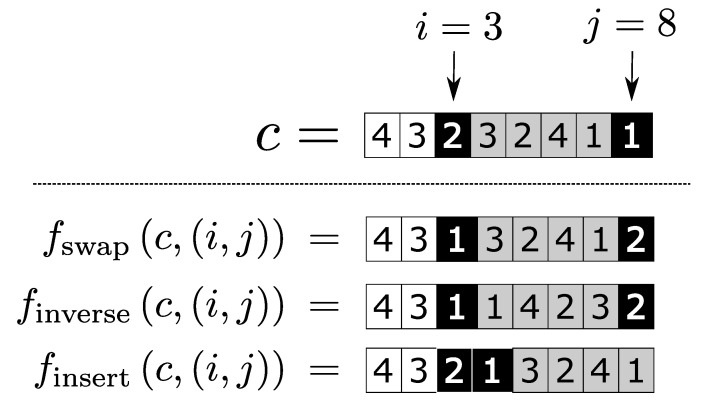
Scheme of mutation functions.

In Figure 3, the comparative operation of the considered mutation functions for the discussed example is presented.

#### 4.5.2. Local Search Mutation Operator

As mutation operator it is proposed to generalize local search operator of Reference [6] as a variation of Reference [22] mutation operator. Thus, for each individual generated in the crossover operator, one mutation function of $F_{\mathrm{mut}}$ is chosen randomly and applied successively $R_{m}$ times in randomly chosen coordinates of the chromosome keeping the beneficial modifications and proceeding with the mutation method from them, giving rise to a mutant population with the same number of individuals as the child population. However, in order to maintain the traditional characteristics of the mutation operator, which is to cause chromosome disturbance regardless of the presence of process improvement or worsening, the option of apply just one execution of a mutation function corresponding to a simple mutation was added in a percentage of individuals. Therefore, only a percentage $\epsilon_{\mathrm{LS}}$ of the population of children is mutated in a local search form, and a percentage $1-\epsilon_{\mathrm{LS}}$ is given a simple mutation. The scheme is presented in Algorithm 2.

The main purpose of this procedure is to perform thorough searches in regions close to known good solutions, as such solutions have been determined to be better than their respective parents and likely to enhance the solutions of previous generations.

The local search and mutation operator of Reference [6] is a special case of our local search mutation operator if we define $R_{m}=N\cdot M$ and $F_{\mathrm{mut}}=\left\{f_{\mathrm{swap}},f_{\mathrm{inverse}},f_{\mathrm{insert}}\right\}$. Thus, our methodology consists of a proposal to generalize the tool of Reference [6] with respect to the mutation. This modification, however simple, may be able to save processing on low complexity JSSP instances by setting a small value for $R_{m}$ and a set of mutation functions with few elements. Moreover, this generalization makes the methodology more versatile, since for high complexity instances, we can define $R_{m}$ as a high value and $F_{\mathrm{mut}}$ as a set with more elements in order to improve the search capability of the proposed technique.

**Algorithm 2** Proposed mutation operator.
**Input:**

*P*_child_
Set of individuals generated using Algorithm 1
*F*
Fitness function$F_{\mathrm{mut}}$ = { *f*_mut,1_, *f*_mut,2_, ..., *f*_mut,*n*_mut__}Set of crossover functions
$\epsilon_{\mathrm{LS}}$
Usage of local search strategy (in %)
*R_m_*
Max mutations for each pair*N* × *M*JSSP instance dimension1: *f*_mut_:= pick_randomly ({*f*_mut,1,_
*f*_mut,2, …,_
*f*_mut,*n*mut_}) 
▷ Take a mutation function randomly
2: *P*_mut_ := {}3:**for**
*c* ∈ *P*_child_
**do**
▷ All the offsprings generated in the multi-crossover operator will be mutated
4:       if rand([0, 1]) ≤ $\epsilon_{\mathrm{LS}}$
**then**
▷ Will there be a local search?
5:              *Fc* := *F(c)*6:             **for**
*i*
**:=** 1 to *R_m_*
**do**

▷ Run exactly Rm perturbations on the considered child *c*
7:                    *r*_1_ := pick_randomly ({1, 2, ..., *N* · *M*})
▷ Choose the coordinates to use in the mutation


   function
8:                    *r*_2_ := pick_randomly ({1, 2, ..., *r*_1_ − 1, *r*_1_ + 1, ..., *N* · *M*})9:                    $\hat{c}$ := *f*_mut_ (*c,* (*r*_1_*, r*_2_))
▷ Apply the mutation function
10:                  *F*_$\hat{c}$_ := *F*($\hat{c}$) 
▷ Evaluate the mutant child
11:                  **if**
*F_$\hat{c}$_* ≦ *Fc*
**then**
▷ If the mutation was beneficial, then
12:                                   *c := $\hat{c}$*

▷ Update the child *c* and continue from it on the next perturbation
13:                                   *Fc* := *F_$\hat{c}$_*14:                  **end if**15:             **end for**16:         **else**
▷ Do not use local search strategy and just perturb the child one time
17:             *r*_1_ := pick_randomly ({1, 2, ..., *N* · *M*})
▷ Choose the coordinates to use in the mutation
        function18:             *r*_2_ := pick_randomly ({1, 2, ..., r_1_ − 1, r_1_ + 1, ..., *N* · *M*})19:             *c* := *f*mut (*c*, (*r*_1_, *r*_2_)) 
▷ Apply the mutation function just one time
20:         **end if**21:         *P*_mut_ := *P*_mut_ ∪ {*c*}|
▷ Update the set of mutant individuals
22: **end for**
**Output:***P*_mut_ Population of mutant individuals

### 4.6. Proposed Massive Local Search Operator

In this work, we propose as massive local search operator an improvement of the elite local search proposed in Reference [6]. This enhancement is through the use of more than one perturbation function, causing the operator to eventually use distinct mutation functions instead of just one, as in Reference [6]. A massive local search operator has as its primary objective to evaluate which disturbances made with respect to some mutation function improve an individual’s fitness. The main purpose of this procedure is to perform thorough searches in regions close to known good solutions, as such solutions have been determined to be better than their respective parents and likely to enhance the solutions of previous generations. This procedure is performed taking into consideration all possible combinations within the coordinates of a good individual using different perturbation strategies. In Reference [6], this procedure is performed only with the mutation function $f_{\mathrm{swap}}$ on the best individual in the current population. In our work, we propose that this procedure occurs using different perturbation functions (We will call “perturbation functions” the functions used in this section to facilitate the description so that there is no confusion with the mutation functions of Section 4.5). However, the perturbation functions used in the massive local search operator are defined in the same way as the mutation functions according to Equation (6). in a given set of perturbation functions $F_{\mathrm{pert}}$. In detail, we propose to randomly take a function in $F_{\mathrm{pert}}$, defined in Equation (7), and then carry out all possible perturbations considering the coordinates of an individual using this function so that beneficial perturbations to the individual are always maintained.

(7) $$F_{\mathrm{pert}}=\left\{f_{\mathrm{pert},1},f_{\mathrm{pert},2},\ldots ,f_{\mathrm{pert},n_{\mathrm{pert}}}\right\},$$

where, $f_{\mathrm{pert},i}$ is a function on the form of the Equation (6) for all *i*.

We emphasize, therefore, that our method does not necessarily demand that $F_{\mathrm{pert}}=F_{\mathrm{mut}}$. However, to facilitate the description and execution of the experiments, we will assume that $F_{\mathrm{pert}}=\left\{f_{\mathrm{swap}},f_{\mathrm{inverse}},f_{\mathrm{insert}}\right\}$. In this case, the algorithm not only performs the successive substitution of operations in a given solution, but occasionally, the technique performs successive insertions and inversions, increasing the diversification of the massive local search performed. Suppose that, over the generations, using a set of mutation functions instead of just one function can improve the operator’s ability to explore the search space.

In this work, we propose that the massive search be applied to a group of individuals and not only on the best individual, as is the case with the elite operator. We believe that this generalization can help the method to avoid premature convergence and stagnation of the population around a local optimum. Mathematically, the massive local search must be applied to all individuals of a given set $P_{\mathrm{massive}}$.

For carrying out the experiments, we assume that $P_{\mathrm{massive}}$ is formed by the two best individuals in the population with the restriction that they are different. We intend that two good and different genetic inheritances are maintained in the population in order to help maintain genetic variability.

Thus, the massive local search operator proposed is coded in Algorithm 3.

**Algorithm 3** Proposed multi-crossover operator.
**Input:**

*p*_massive_
Set of individuals selected for massive local search
*F*
Fitness function$F_{\mathrm{pert}}$ = {$f_{\mathrm{pert},1}$, $f_{\mathrm{pert},2}$, …, $f_{\mathrm{pert},n_{\mathrm{pert}}}$}Set of crossover functions*N* × *M*JSSP instance dimension1: *f*_pert_ := pick_-_randomly ({$f_{\mathrm{pert},1}$, $f_{\mathrm{pert},2}$, …, $f_{\mathrm{pert},n_{\mathrm{pert}}}$})
▷ Choose randomly a perturbation function
2: *P*_improved_ = {}3: **for**
*c* ∈ *P*_improved_
**do**
▷ The procedure will massively search around all the individuals in *P*_massive_
4:        *F_c_* := *F*(*c*)
▷ Calculate the initial fitness of the individual *c*
5:        **for**
*i* := 1 to *N*·*M*
**do**
▷ All the combination of the chromosome coordinates will be considered
6:              **for**
*j* := 1 to *N*·*M*
**do**7:                     $\hat{c}$ := $f_{\mathrm{pert}}$ (*c*, (*i*,*j*))
▷ Apply the perturbation considering the coordinates *i* and *j*
8:                     Fc^ := $F(\hat{c})$
▷ Evaluate the fitness of the perturbed individual
9:                      **if**
Fc^ ≦ $F_{c}$
**then**

▷ If the perturbation was beneficial, then
10                          *c* := c^
▷ Update the individual and execute the next perturbations from this version
11:                         $F_{c}$ := Fc^12:                     **end if**13:              **end for**14:        **end for**15:        *P*_improved_ = *P*_improved_ ∪ {*c*}
▷ Update the set with the new improved individual
16: **end for****Output:**       *P*_improved_      Improved individuals

### 4.7. Scheme of Use for Proposed Operators: Algorithm Structure

The use of all operators together follows a methodology similar to that used by Reference [6], which consists of the following central steps:

1 Initiate the configuration of our method, which include choosing the standard definition of parameters and determining the crossover, mutation and perturbation functions;
2 Generate the initial population using the shuffle function defined in Equation (2);
3 Select the individuals and perform our multi-crossover operator;
4 Perform our local search operator on all the offsprings generated on the previous step;
5 Run the massive local search operator to look for better solutions around a group of good individuals;
6 Generate a new population using the roulette wheel strategy retaining the best individual;
7 Return to the first step.

**Figure 4:**
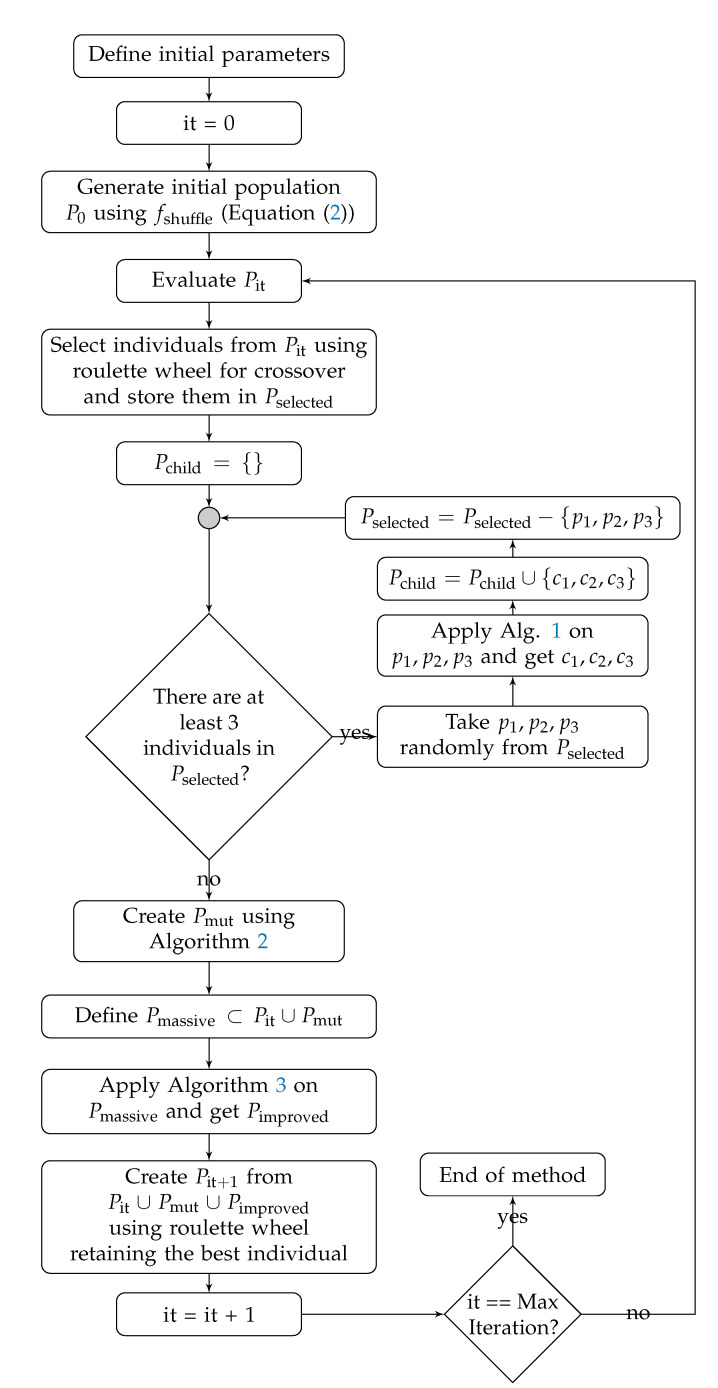
Flow chart of our proposed Multi-Crossover Local Search Genetic Algorithm.

Notice that the method must be used to solve one JSSP instance at a time. Figure 4 shows the flowchart containing all the details about the steps of our proposed mXLSGA, which is the proposed meta-heuristic for application in JSSP instances.

Observing the flowchart of the proposed method, we can see some clear differences between mXLSGA and the techniques that served as inspiration for its making. We can immediately see that the use of multi-crossover strategies with various crossover functions is something unique to the proposed methodology. Furthermore, only mXLSGA uses more than one perturbation function in the massive local search operator and applies them to a set of individuals. In detail, all GA-like methods influenced mXLSGA modeling, however, there are also significant differences, as highlighted in the sequence:

- **Basic GA**:
  - **Inspiration**: The sequence of operations.
  - **Differences**: The mXLSGA uses much more operators such as massive local search operator.
- **GSA** [22]:
  - **Inspiration**: Applications of just one crossover function more than once or until the procedure find an individual better than the worst individual in the population. Successive applications of just one mutation function.
  - **Differences**: The mXLSGA applies various crossover functions more than once or until the method find an individual better than a parent. Successive applications of various mutation functions.
- **LSGA** [4]:
  - **Inspiration**: The mutation operator is composed of two possible procedures: the first performing a local search with successive applications of a variant of the $f_{\mathrm{swap}}$ mutation function; and the second being a simple mutation in the form of a single perturbation.
  - **Differences**: The mXLSGA also has these two possible procedures in its mutation operator. However, it uses a group of mutation functions that the method uses during its iterations in these procedures.
- **aLSGA** [6]:
  - **Inspiration**: We rely mainly on mutation and elite local search operators. The mutation operator performs successive applications of a group of mutation functions on a chromosome. The elite local search operator performs successive perturbations with $f_{\mathrm{swap}}$ considering all possible combinations between the coordinates of the best chromosome in the population.
  - **Differences**: Our mutation operator is divided into two possible subroutines: the first with local search behavior by applying successive perturbations with a group of mutation functions, such as aLSGA; and the second being formed from a simple mutation so that only one perturbation is applied to the chromosome. Unlike aLSGA’s elite local search operator, mXLSGA’s massive local search operator uses a set of perturbation functions, and not just the function $f_{\mathrm{swap}}$, on a set of individuals and not just on the best individual.

In summary, we present in Table 1 how each of the GA-like techniques uses the strategies mentioned and we compare this use with our mXLSGA.

## 5. Implementation and Experimental Results

We demonstrate the effectiveness of the proposed method through three different case studies: the first dedicated to analyzing the operators of the method in isolation to evaluate the influence of each one of them during the solution of JSSP instances; the second specialized in evaluating the capacity of the method to find optimal solutions to the problem addressed, being compared with different types of meta-heuristics that make up the state of the art; and the third dedicated to comparing our method with other GA-like techniques when solving the JSSP.

The proposed algorithm was coded using Matlab software and the tests were performed on a computer with 2.4 GHz Intel(R) Core i7 CPU and 16 GB of RAM. We emphasize that we only used the standard functions of the Matlab IDE to conduct the computational experiments and we do not use ready-made optimization software packages.

### 5.1. Case Study I: Analysis of Isolated Operators

In this case study, we evaluated different configurations of the method to investigate how each of the operators proposed in this work acts on our method. Specifically, we want to observe which operators most strongly influence the proposed method. For this, we evaluated different configurations of our technique in three LA [17] instances. These configurations were stipulated so that we were able to analyze the influence of each of the operators separately. That is, we defined a configuration as a basic GA, another as a basic GA with the multi-crossover operator, another as a basic GA with a version of the proposed local search operator, and so on. For this, we will investigate the operation of the following configurations:

- **GA**: Basic GA;
- **GA+mX**: Basic GA with proposed multi-crossover operator from Section 4.4;
- GA+LS*: Basic GA with proposed local search operator from Section 4.5 using $\epsilon_{\mathrm{LS}}=1$, i.e., performing local search on all offsprings that are generated at the crossover operator;
- GA+LS**: Basic GA with proposed local search operator from Section 4.5 using $\epsilon_{\mathrm{LS}}=0.8$, i.e., performing local search on 80% of all offsprings that are generated at the crossover operator;
- **GA+ELSA**: Basic GA with simple mutation (just one use of $f_{\mathrm{swap}}$) and using proposed massive local search operator with $F_{\mathrm{pert}}=\left\{f_{\mathrm{swap}}\right\}$ (elite local search of Reference [6]);
- **GA+MLS**: Basic GA with simple mutation (just one use of $f_{\mathrm{swap}}$) and using proposed massive local search operator with $F_{\mathrm{pert}}=\left\{f_{\mathrm{swap}},f_{\mathrm{inverse}},f_{\mathrm{insert}}\right\}$;
- GA+mX+LS**: Using our multi-crossover operator in GA+LS** previous configuration;
- **mXLSGA**: Proposed method with all proposed operators (Figure 4).

The details of the configuration of each of the evaluated algorithms are shown in Table 2.

**Figure 5:**
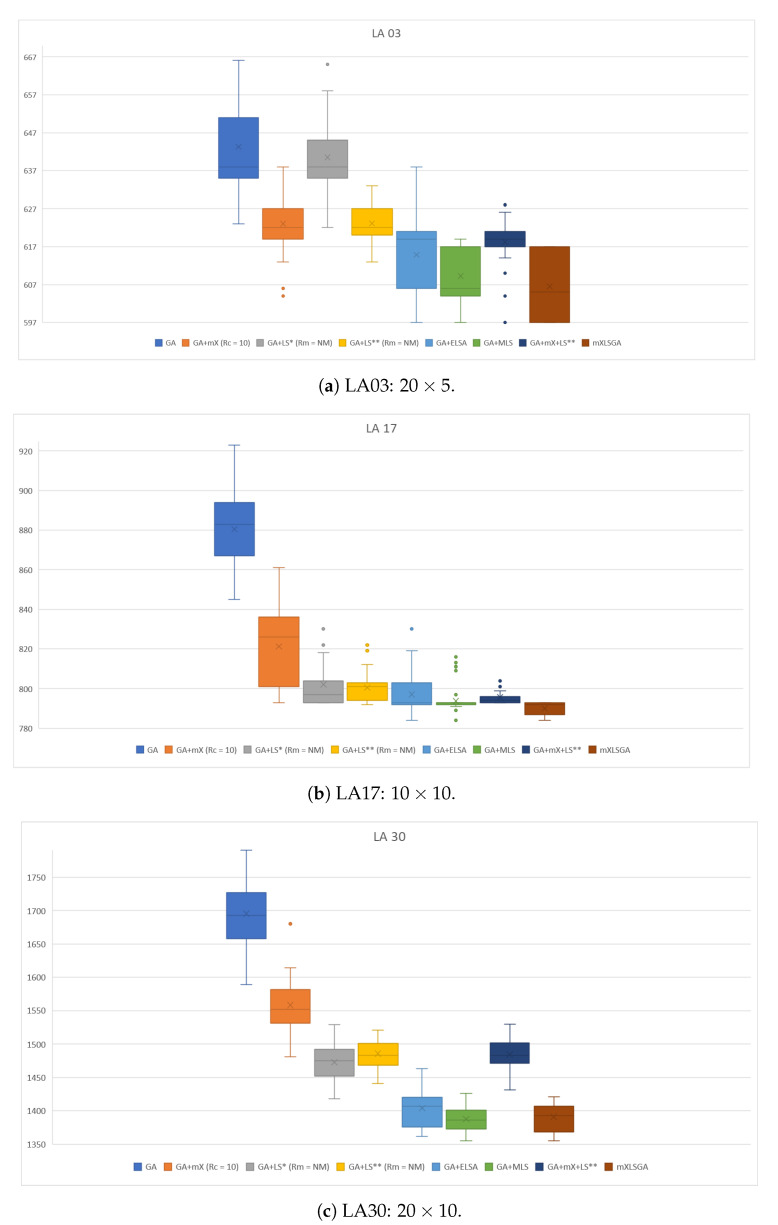
Box plots of the fitness values of 35 executions of the different configuration of our method.

Each of the configurations presented in this case study was executed 35 times in three LA instances of different sizes: LA 03, with a dimension of 20×5, considered easy level; LA 17, of size 10×10, of medium level; and LA 30, with a size of 20×10, of difficult level. These instances were defined so that at least one instance of each of the three main groups of dimensions was considered to verify the efficiency of the algorithm and its behavior in instances of varying dimensions, from the simpler to the most complex. The fitness values for each configuration were used to make the box plots in Figure 5.

Looking at the box plots shown in Figure 5, we can see that the addition of any of the operators proposed in GA was very beneficial. However, some operators have greater influence in certain cases. For example, the multi-crossover operator has greater influence on less complex bases, since in Figure 5a it works as well as or even better than the GA+LS* and GA+LS** configurations, while on more complex bases (Figure 5b,c) the GA+mX configuration presents results statistically inferior to the results of GA+LS* and GA+LS**.

Concerning mutation operators that use local search strategies, we noticed that as the complexity of the analyzed instance increases, the performance of GA+LS* improves in relation to the GA+LS** configuration. This is because, the greater the complexity of the instance, the use of local search strategies becomes more advantageous than the genetic variability guaranteed by the operator with the use of $\epsilon_{\mathrm{LS}}\neq 1$. However, we can see in Figure 5a that, in instances of less complexity, the GA+LS** configuration presents much better and more stable results than those presented by GA+LS*. Thus, it is a good strategy to use a value between 0.8 and 1 for $\epsilon_{\mathrm{LS}}$ in the next case studies.

We can also observe that the massive local search operator is the operator that present better quality and greater stability in the results. The addition of massive local search operators in basic GA results in better fitness values compared to the addition of two distinct operators, as is the case with the GA+mX+LS** configuration, which results in lower fitness values than GA+ELSA and GA+MLS configurations in the box plots of Figure 5a,c. Also, the use of more than one disturbance function in $F_{\mathrm{pert}}$ has resulted in GA+MLS presenting better and more stable results than the results of GA+ELSA, which uses only the $f_{\mathrm{swap}}$ function in $F_{\mathrm{pert}}$, in all evaluated cases. Thus, we can conclude that the operator with the greatest influence is the massive local search operator.

The use of two local search operators in GA + mX + LS** made this configuration more stable than the simplest configurations that use only one operator, in this case the configurations GA + mX, GA + LS* and GA + LS**. In addition, also compared to these configurations, GA + mX + LS** presents better fitness values in the instances LA03 (Figure 5a) and LA30 (Figure 5c).

Finally, the configuration of the proposed methodology that corresponds to the joint use of all the operators discussed is the mXLSGA method, which presents the best results in all evaluated cases, with greater accent in the LA03 instances (Figure 5a) and LA17 (Figure 5b). This confirms that the use of all operators concurrently is the best configuration for the methodology since it is in this configuration that the best results are obtained.

Table 3 shows the average time required considering 35 independent executions for all the proposed method configurations to solve the JSSP instances in this case study. As we can see, basic GA is the fastest technique of all compared. However, we note that the addition of the multi-crossover operator (GA + mX) does not radically compromise the computational time, but, as shown in the box-plots of Figure 5a–c, this addition considerably improves the performance of the method in obtaining good results. In the case of versions that use only massive local search operators (GA + ELSA and GA + MLS), it is clear that the performance is improved and that, in smaller instances such as LA03 and LA17, the computational time is not drastically increased. However, that is not the case of the LA30 instance. This is due to the quadratic behavior of these operators, since, as we can see in their formulation in Algorithm 3, in each iteration of the method occurs ${\left(N\cdot M\right)}^{2}$ perturbations in the chromosomes from $P_{\mathrm{massive}}$. Something similar occurs with configurations that only use local search strategies in the mutation operator (GA + LS* and GA + LS**). Specifically, we can see that these are the most expensive isolated operators in time cost in the case of smaller instances (LA03 and LA17) and this cost increases proportionally according to the complexity and size of the instance. These facts are reflected in the GA + mX + LS** configuration, whose average time in all instances is approximately the sum of the times of the GA + mX and GA + LS** configurations. In all instances, the configuration that obtains the best performance (mXLSGA) is also the most costly in computational time, but this is an expected result since this technique uses all the operators described in this work. Thus, in the following case studies, we will direct our analysis and comparisons to our mXLSGA.

### 5.2. Case Study II: Mxlsga for JSSP and Comparison with Other Algorithms

In this case study, we will evaluate the capacity of the proposed methodology to find optimal solutions in the search space. For this, we intend to demonstrate the effectiveness of the method when applied in JSSP instances present in the specialized literature.

In details, to evaluate the proposed approach, experiments were performed in 58 JSSP instance scenarios, 3 FT instances [16], 40 LA instances [17], 10 ORB instances [18] and 5 ABZ instances [19]. The results obtained in the execution of the tests were compared with papers from the specific literature. The articles determined for each comparison were selected because they are relevant works in the literature, which deal with the JSSP with the same specific instances and, when existing, papers published in the last three years were adopted. The papers selected for comparison of results were as follows: SSS [61], GA-CPG-GT [14], DWPA [15], GWO [11], HGWO [12], MA [9], IPB-GA [8] and aLSGA [6].

The configuration of parameters for the mXLSGA was established through tests and also taking into consideration, when possible, a closer parameterization of the works that were used for comparison. In this way, the parameters were defined as shown in Table 4.

The proposed mXLSGA method was executed 10 times for each JSSP instance and the best value obtained was used for comparison with other papers. In most of the comparative works, the authors do not mention the processing time of their techniques and only present the best result. We proceed in the same way for this case study, reserving a more detailed analysis using time and other statistical measures for the next case study, in which we programmed a version of other compared techniques and, therefore, we were able to observe such measures.

Table 5 shows the results derived from the LA [17], FT [16], ORB [18] and ABZ [19] instance tests. The columns indicate, respectively, the instance that was tested, the instance size (number of Jobs × number of Machines), the optimal solution of each instance, the results achieved by each method (best solution found and error percentage (Equation (8)), and the mean of the error for each benchmark (MErr).

(8) $$E_{\%}=100\times \frac{\mathrm{Best}-\mathrm{BKS}}{\mathrm{BKS}},$$

where “E%” is the relative error, “BKS” is the best known Solution and “Best” is the best value obtained by executing the algorithm for each instance.

As shown in Table 5, mXLSGA found the best known solution in 100% of FT instances, 70% of LA instances, 30% of ORB instances, and 40% of ABZ instances.

The mXLSGA proposal reached in 28 LA instances the best known solution and obtained a mean relative error (MErr) of 0.61. The SSS and HGWO methods obtained a lower average error than our mXLSGA, assuming 0.59 and 0.38, respectively, but they did not test all the LA instances. If we only consider the instances that have been tested by SSS, our mXLSGA proposal would obtain a MErr of 0.46. If we only consider the instances tested by HGWO, our method would get 0.00 from MErr. Specifically, in FT instances, the mXLSGA reached in 3 instances the best known solution and obtained a MErr of 0.00. In ORB instances, the mXLSGA reached in 3 instances the best known solution and obtained a MErr 0.54, it is the method with the lowest MErr of all compared algorithms. In ABZ instances, the mXLSGA reached in 2 instances the best known solution and obtained a MErr of 4.46. The method that achieved a minor error was GA-CPG-GT, but this work did not test in all ABZ instances, and if we compare only the instances that GA-CPG-GT was tested, mXLSGA would have gotten 0.00 relative mean error, that is, the mXLSGA achieved the best known solution in the first 2 ORB instances.

In particular, our method surpassed the technique on which it was based, which in this case is aLSGA. In LA instances, our method got 6 BKS more than aLSGA.The aLSGA obtained in the LA instances a MErr of 0.80 and mXLSGA obtained a MErr of 0.61, but aLSGA was tested only in the first 35 LA instances, if we consider the MErr only for the 35 LA instances, mXLSGA would get a MErr of 0.37, which is less than half the value obtained by aLSGA. For ORB instances, mXLSGA obtained a MErr less than one third of the one obtained by aLSGA. The improvement achieved by mXLSGA is certainly due to the insertion of the multi-crossover operator and the enhancements employed in local search techniques.

Analyzing the data presented in Table 5, we can see that in the tested JSSP instances, the proposed mXLSGA results better or equal to the compared state-of-the-art algorithms.

### 5.3. Case Study III: Statistical Analysis of Ga-Like Methods

This case study was designed and executed to verify the effectiveness of the mXLSGA method when compared to the main GA-like methods that build the specialized literature: the basic GA, GSA [22], LSGA [4], aLSGA [6]. We compared mXLSGA with such techniques because they were the basis of inspiration for its modeling and because they still represent the state of the art in GA-based metaheuristics to solve the JSSP. The effectiveness must be guaranteed through the analysis of different statistical measures and the time of each method. Indeed, we implemented all the compared techniques to perform the analyzes. So that all the methods were coded as faithfully as possible to the descriptions present in their respective articles, however, we emphasize that the coding may differ from the original coding for several reasons, such as: different programming techniques; parameterization of the method, for example, the number of executions for the method; some detail of the method that is not present in the text of the original work or that we have interpreted differently; and so forth.

We try to follow the parameterization as faithful as possible to the original parameterization of each technique, however, we use 100 individuals and 100 iterations in all the considered methods. In details, the configuration used is presented in Table 6.

The compared techniques were executed 35 times on instances of different dimensions in which our method can find the optimal value of makespan considering case study II (Section 5.2). In Table 7, we can see some statistical information about these executions, namely: the best fitness value achieved by the technique (Best); the worst fitness value achieved (Worst); the average of the fitness values of the executions of each technique (Average); the standard deviation of these values (SD); the number of times the method has reached the optimal value (Number opt); the number of iterations (Number it) needed to reach the optimum value; and the average time (AT) in seconds that the technique takes to perform 100 iterations.

As shown in Table 7, it is noteworthy that mXLSGA found the optimal fitness value in all instances analyzed, and in instances FT 06, LA 01, LA 06 and LA 11, the proposed method found the optimal value in all executions. The mXLSGA presented the lowest worst value and the lowest average in all instances. In the instance LA 16, LA 23, and LA 26, mXLSGA did not obtain the lowest standard deviation value, however, it was the only method that found the optimal value in all these instances. Our method was the method that required the longest average execution time, but as it will be better explained in the following paragraphs, this is not in any way a deficiency in our method, as it does not need all 100 generations to converge.

**Figure 6:**
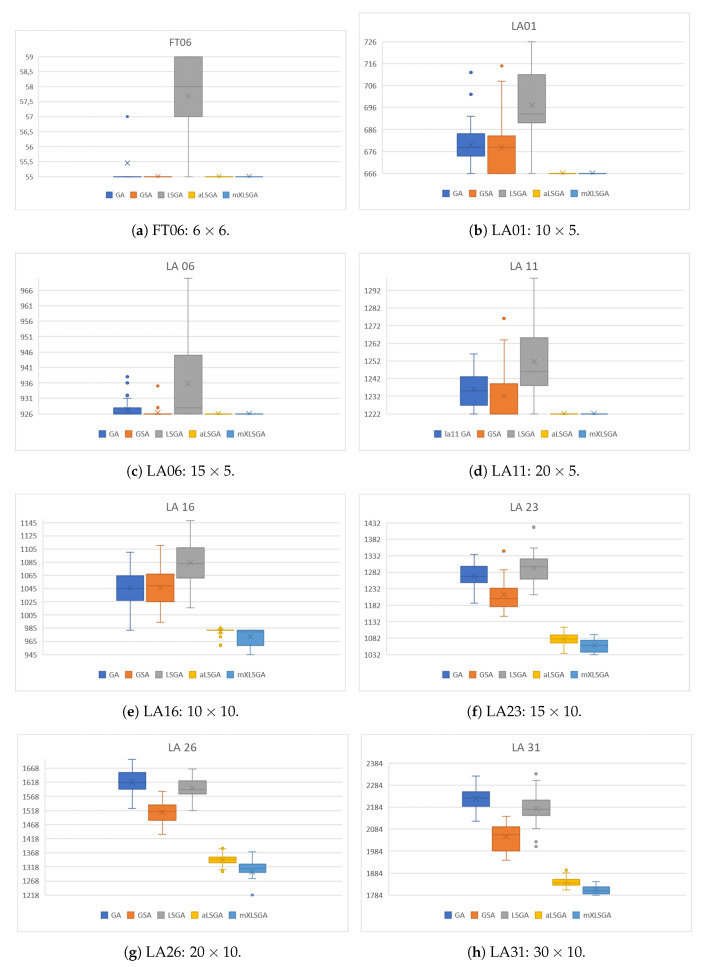
Box plots of the fitness values of 35 executions of the GA-Like methods.

To visualize the statistical performance of each method, we made the box plots of the fitness values achieved in this case study, which are shown in Figure 6. As highlighted in the case study I (Section 5.2), box plots make it clear how the presence of a massive local search operator makes the method much more robust compared to the others, since in all the instances the aLSGA and mXLSGA methods are the most stable and have the best fitness values. This difference is even accentuated as the complexity of the instance increases. Also, the graphs reflect the values presented in Table 7, since mXLSGA is the box below the other boxes in all evaluations. We also noticed some details, such as why the mXLSGA standard deviation is the largest when executed in the LA 26 instance since this is because the technique finds the optimum value 6 times as a discrepancy. It is also clear from the graph that, statistically, our method is the method that most finds the best makespan settings.

**Figure 7:**
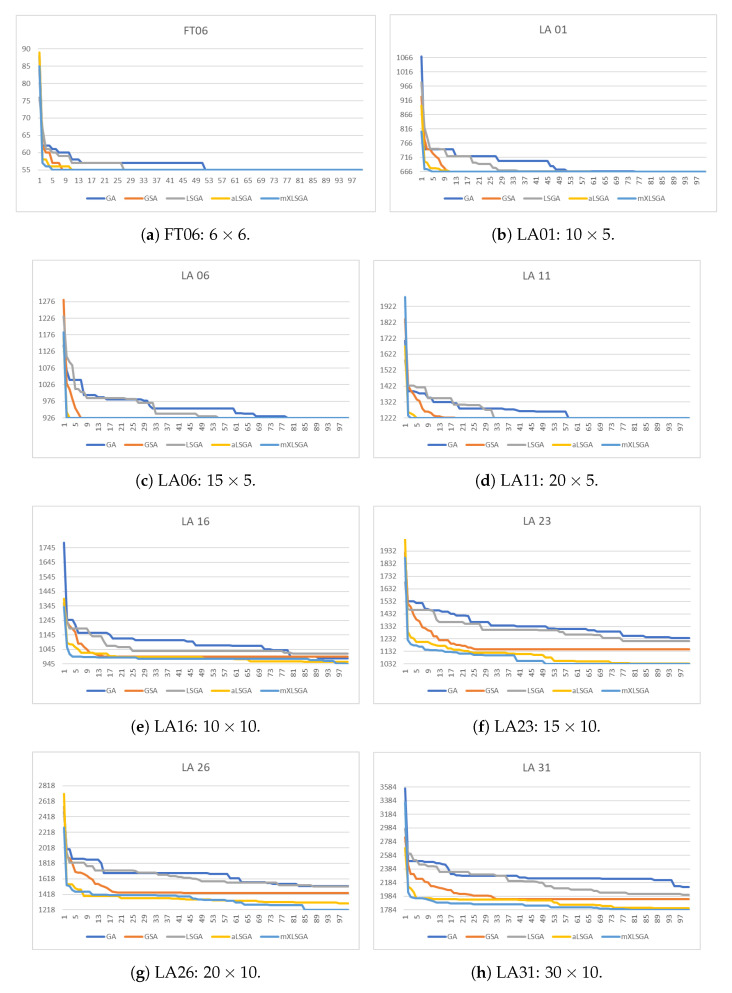
Convergence curves of GA-like methods over 100 generations.

In Figure 7, we highlight the convergence curves of the fitness value of the best individual of the 35 executions of each method in each instance during the 100 generations. It is clear that, as the complexity of the assessed instance increases, the number of iterations that each method needs to achieve the best value also increases. However, in none of the situations did our method requires all the 100 generations to find the best value. Indeed, mXLSGA finds the optimal value of makespan, or very close to optimum, with approximately 50 generations in more difficult instances. Whereas, in the case of simpler instances, such as FT 06 (Figure 7a), LA 01 (Figure 7b), LA 06 (Figure 7c) and LA 11 (Figure 7d), we realize that our method achieves optimal fitness before the 5th iteration. This fact does not occur with the other evaluated techniques, which need more iterations to reach the optimal value or, with the configuration used, they were not able to reach any of the optimum points, as was the case with all techniques with exception of mXLSGA in instances LA 16 (Figure 7e), LA 23 (Figure 7f), LA 26 (Figure 7g) and LA 31 (Figure 7h). In this way, time is no longer a major concern for our method, since it requires only 50% of the iterations used to achieve good results in larger instances, which reduces the time spent in half, or it only needs 5% of iterations to achieve the optimum in simpler instances.

In the sequence, still concerning the computational time analysis, we will evaluate the following situation: in each instance considered in this case study we will execute all the techniques taking as a stopping criterion the computational time instead of the maximum number of iterations. That is, all techniques will be performed for the same amount of time. In this way, for each instance, we will take as a time limit for all techniques the time it took the most time-consuming method to complete 100 iterations with respect to that instance according to the Table 7. For example, for this experiment, all methods have 39.78 seconds to search for the optimal value of the instance FT-06, since this amount of time is the same amount that the GSA technique, which is the most time-consuming method in this instance, takes to perform 100 iterations on this JSSP instance. All techniques were performed independently 35 times following the strategy described. The results are summarized in Table 8.

In Table 8, we can see that there was some improvement in all the techniques that had more time to be performed. A clear example is basic GA, which showed a performance improvement in practically all measures considering all instances. However, the complexity of the evaluated instance remains a predominant factor with respect to the performance of the technique in this experiment, since in the case of a less complex instance such as FT06, all the evaluated methods were able to find the optimal value 55 in all the 35 executions. In the case of more complex instances such as instances LA16, LA23, LA26, and LA31 only the proposed method was able to find the optimal value, even with all techniques being able to be executed with the same amount of time. In addition, also in these instances, our method presented without a tie the best statistical measures of best fitness value, worst fitness value, and average fitness. In other words, our methodology presented the best performance in more complex instances and tied these measures in simpler instances in this experiment. This serves as an indication that the methodology proposed in this work provides better searchability for the technique, making it more efficient and surpassing other GA-like algorithms present in the literature.

## 6. Conclusions

The objective of this work was to develop an approach to optimize the makespan in the job shop scheduling instances. The proposed technique for achieving the goal was a GA with improved local search techniques and a multi-crossover operator. To evaluate the proposed approach, experiments were conducted in three different case studies.

In the first case study, all operators of mXLSGA were individually evaluated. It was found that all operators jointly corroborate the method for improving the results obtained. That is, no single operator obtained better results than the complete method by all operators. However, we can observe that the most influential operator is the massive search operator, which has greater search power than the modified mutation and multi-crossover operators that we propose.

By analyzing the results obtained in the second case study, we can observe that the proposed method achieves competitive results in JSSP instances and it is able to find good makespan results. The mXLSGA obtained competitive MErr with respect to the results achieved by the compared algorithms in the LA, ORB and ABZ instances. In the FT instance mXLSGA got 0.00% error and tied with the aLSGA algorithm. Through the analysis of the results we can see that mXLSGA is a competitive and versatile method that achieves good results in instances of varying complexity.

In the last case study, we compared our method with the techniques on which its modeling was based. In this case, the GA-like techniques that make up the state of the art in JSSP solution with GA-based meta-heuristics. In this case study, our method obtained the best statistical measures compared to the other techniques. However, the computational time used to obtain these results considering 100 iterations for each method was the largest for mXLSGA. In addition, in this case study we show that, according to the convergence curves, our method needs only 5% of all iterations to achieve the optimal solution in small instances, and only half of the iterations to obtain the optimal solution or very close to the optimal solution in larger instances. The fact that does not occur with the other compared techniques. Thus, time is not a concern for mXLSGA, as it works well even with the use of less demanding configurations. We finished this case study with an experiment in which all GA-like methods could be executed during the same amount of time according to each JSSP instance considered and, observing the results, we concluded that the proposed approach presents better performance than the others GA-based approaches, especially when considering more complex instances.

Analyzing the three case studies presented in this paper, we conclude that mXLSGA is a robust method, with the ability to obtain good results in instances of different complexities and that it has a faster convergence rate when compared to other GA-like methods. The mXLSGA presents better or at least competitive results when compared to other meta-heuristics found in the specialized literature.

In future works, we will study the feasibility of the method in similar combinatorial optimization problems, such as flexible job shop scheduling problem, flow shop scheduling problem, and so forth. Also, we will consider the inclusion of adaptive rules in the discussed operators to control the auto-configuration of parameters. For example, in a future version of our mXLSGA, the method should automatically adjust the crossover and the mutation rates during the iterations according to its performance.

## Figures and Tables

**Table 1:** Comparison between the use of different strategies by each method. “Yes” means that the method uses the corresponding strategy and “No” means that it does not.

Strategy\Method	GA	GSA	LSGA	aLSGA	mXLSGA
Set of crossover functions (multi-crossover)	No	No	No	No	Yes
Search area adaptation on crossover	No	Yes	No	No	Yes
Local search on mutation	No	Yes	Yes	Yes	Yes
Set of mutation functions	No	No	No	Yes	Yes
Eventually simple mutation (just one application)	Yes	No	Yes	No	Yes
Massive local search	No	No	No	Yes	Yes
Set of perturbation functions	No	No	No	No	Yes

**Table 2:** Set up for case study I. Where $c_{\mathrm{best}}$ is the best individual in the population and $c_{\mathrm{best},1}$ and $c_{\mathrm{best},2}$ are the two best individuals in the population who are different.

	GA	GA+mX	GA+LS*	GA+LS**	GA+ELSA	GA+MLS	GA+mX+LS**	mXLSGA
Population size	100	100	100	100	100	100	100	100
Generations	100	100	100	100	100	100	100	100
Crossover rate	0.95	0.95	0.95	0.95	0.95	0.95	0.95	0.95
Mutation rate	0.05	0.05	1	1	0.05	0.05	1	1
$R_{c}$	1	10	1	1	1	1	10	10
$R_{m}$	1	1	N·M	N·M	1	1	N·M	N·M
$F_{\times }$	$\left\{\mathrm{PMX}\right\}$	$\left\{\mathrm{OX}2,\mathrm{PMX}\right\}$	$\left\{\mathrm{PMX}\right\}$	$\left\{\mathrm{PMX}\right\}$	$\left\{PMX\right\}$	$\left\{\mathrm{PMX}\right\}$	$\left\{\mathrm{OX}2,\mathrm{PMX}\right\}$	$\left\{\mathrm{OX}2,\mathrm{PMX}\right\}$
$F_{\mathrm{mut}}$	$\left\{f_{\mathrm{swap}}\right\}$	$\left\{f_{\mathrm{swap}}\right\}$	$\left\{f_{\mathrm{swap}},f_{\mathrm{inverse}},f_{\mathrm{insert}}\right\}$	$\left\{f_{\mathrm{swap}},f_{\mathrm{inverse}},f_{\mathrm{insert}}\right\}$	$\left\{f_{\mathrm{swap}}\right\}$	$\left\{f_{\mathrm{swap}}\right\}$	$\left\{f_{\mathrm{swap}},f_{\mathrm{inverse}},f_{\mathrm{insert}}\right\}$	$\left\{f_{\mathrm{swap}},f_{\mathrm{inverse}},f_{\mathrm{insert}}\right\}$
$F_{\mathrm{pert}}$	{}	{}	{}	{}	$\left\{f_{\mathrm{swap}}\right\}$	$\left\{f_{\mathrm{swap}},f_{\mathrm{inverse}},f_{\mathrm{insert}}\right\}$	{}	$\left\{f_{\mathrm{swap}},f_{\mathrm{inverse}},f_{\mathrm{insert}}\right\}$
$\epsilon_{\mathrm{LS}}$	0	0	1	0.8	0	0	0.8	0.8
$P_{\mathrm{massive}}$	{}	{}	{}	{}	$\left\{c_{\mathrm{best}}\right\}$	$\left\{c_{\mathrm{best}}\right\}$	{}	$\left\{c_{\mathrm{best},1},c_{\mathrm{best},2}\right\}$

**Table 3:** Average time spent in seconds by each configuration of the proposed method to solve each instance of the Job Shop Scheduling Problem (JSSP) during 35 independent executions.

	LA03	LA17	LA30
GA	3.60	4.09	5.95
GA+mX	5.40	6.21	9.62
GA+LS*	20.01	37.70	115.78
GA+LS**	16.68	30.23	100.50
GA+ELSA	9.45	28.35	177.28
GA+MLS	9.62	29.23	184.76
GA+mX+LS**	20.34	34.87	106.14
mXLSGA	32.42	70.29	236.32

**Table 4:** Configuration of parameters for our Multi-Crossover Local Search Genetic Algorithm (mXLSGA).

Population size	100
Maximum number of generations	100
Crossover probability	0.95
Mutation probability	0.95
$\epsilon_{\mathrm{LS}}$	0.95
$R_{c}$	10
$R_{m}$	2·N·M
$F_{\times }$	$\left\{\mathrm{OX}2,\mathrm{PMX}\right\}$
$F_{\mathrm{mut}}$	$\left\{f_{\mathrm{swap}},f_{\mathrm{inverse}},f_{\mathrm{insert}}\right\}$
$F_{\mathrm{pert}}$	$\left\{f_{\mathrm{swap}},f_{\mathrm{inverse}},f_{\mathrm{insert}}\right\}$
$P_{\mathrm{massive}}$	Two best and different individuals in the population

**Table 5:** Comparison of computational results between mXLSGA and other algorithms. The symbol “-” means “no evaluated in that instance”.

Instance	Size	BKS	mXLSGA		SSS		GA-CPG-GT		DWPA		GWO		HGWO		MA		IPB-GA		aLSGA	
			Best	$E_{\%}$	Best	$E_{\%}$	Best	$E_{\%}$	Best	$E_{\%}$	Best	$E_{\%}$	Best	$E_{\%}$	Best	$E_{\%}$	Best	$E_{\%}$	Best	$E_{\%}$
LA01	10×5	666	666	0.00	666	0.00	666	0.00	666	0.00	666	0.00	666	0.00	666	0.00	666	0.00	666	0.00
LA02	10×5	655	655	0.00	655	0.00	655	0.00	655	0.00	655	0.00	655	0.00	655	0.00	655	0.00	655	0.00
LA03	10×5	597	597	0.00	597	0.00	597	0.00	614	2.84	597	0.00	597	0.00	597	0.00	599	0.33	606	1.50
LA04	10×5	590	590	0.00	590	0.00	590	0.00	598	1.35	590	0.00	590	0.00	590	0.00	590	0.00	593	0.50
LA05	10×5	593	593	0.00	593	0.00	593	0.00	593	0.00	593	0.00	593	0.00	593	0.00	593	0.00	593	0.00
LA06	15×5	926	926	0.00	926	0.00	926	0.00	926	0.00	926	0.00	926	0.00	926	0.00	926	0.00	926	0.00
LA07	15×5	890	890	0.00	890	0.00	890	0.00	890	0.00	890	0.00	890	0.00	890	0.00	890	0.00	890	0.00
LA08	15×5	863	863	0.00	863	0.00	863	0.00	863	0.00	863	0.00	863	0.00	863	0.00	863	0.00	863	0.00
LA09	15×5	951	951	0.00	951	0.00	951	0.00	951	0.00	951	0.00	951	0.00	951	0.00	951	0.00	951	0.00
LA10	15×5	958	958	0.00	958	0.00	958	0.00	958	0.00	958	0.00	958	0.00	958	0.00	958	0.00	958	0.00
LA11	20×5	1222	1222	0.00	1222	0.00	1222	0.00	1222	0.00	1222	0.00	1222	0.00	1222	0.00	1222	0.00	1222	0.00
LA12	20×5	1039	1039	0.00	-	-	1039	0.00	1039	0.00	1039	0.00	1039	0.00	1039	0.00	1039	0.00	1039	0.00
LA13	20×5	1150	1150	0.00	-	-	1150	0.00	1150	0.00	1150	0.00	1150	0.00	1150	0.00	1150	0.00	1150	0.00
LA14	20×5	1292	1292	0.00	-	-	1292	0.00	1292	0.00	1292	0.00	1292	0.00	1292	0.00	1292	0.00	1292	0.00
LA15	20×5	1207	1207	0.00	-	-	1207	0.00	1273	5.46	1207	0.00	1207	0.00	1207	0.00	1207	0.00	1207	0.00
LA16	10×10	945	945	0.00	947	0.21	946	0.10	993	5.07	956	1.16	959	1.48	946	0.10	946	0.10	946	0.10
LA17	10×10	784	784	0.00	-	-	784	0.00	793	1.14	790	0.76	784	0.00	784	0.00	784	0.00	784	0.00
LA18	10×10	848	848	0.00	-	-	848	0.00	861	1.53	859	1.29	857	1.06	858	1.17	853	0.58	848	0.00
LA19	10×10	842	842	0.00	-	-	842	0.00	888	5.46	845	0.35	845	0.35	-	-	866	2.85	852	1.18
LA20	10×10	902	902	0.00	-	-	907	0.55	934	3.54	937	3.88	946	4.87	-	-	913	1.21	907	0.55
LA21	15×10	1046	1059	1.24	1076	2.86	1090	4.20	1105	5.64	1090	4.20	-	-	1081	3.34	1081	3.34	1068	2.10
LA22	15×10	927	935	0.86	-	-	954	2.91	989	6.68	970	4.63	-	-	954	2.91	970	4.63	956	3.12
LA23	15×10	1032	1032	0.00	-	-	1032	0.00	1051	1.84	1032	0.00	-	-	1032	0.00	1032	0.00	1032	0.00
LA24	15×10	935	946	1.17	-	-	974	4.17	988	5.66	982	5.02	-	-	976	4.38	1002	7.16	966	3.31
LA25	15×10	977	986	0.92	-	-	999	2.25	1039	6.34	1008	3.17	-	-	999	2.25	1023	4.70	1002	2.55
LA26	20×10	1218	1218	0.00	-	-	1237	1.55	1303	6.97	1239	1.72	-	-	-	-	1273	4.51	1223	0.41
LA27	20×10	1235	1269	2.75	1256	1.70	1313	6.31	1346	8.98	1290	4.45	-	-	-	-	1317	6.63	1281	3.72
LA28	20×10	1216	1239	1.89	-	-	1280	5.26	1291	6.16	1263	3.86	-	-	-	-	1288	5.92	1245	2.38
LA29	20×10	1152	1201	4.25	-	-	1247	8.24	1275	10.67	1244	7.98	-	-	-	-	1233	7.03	1230	6.77
LA30	20×10	1355	1355	0.00	-	-	1367	0.88	1389	2.50	1355	0.00	-	-	-	-	1377	1.62	1355	0.00
LA31	30×10	1784	1784	0.00	1784	0.00	1784	0.00	1784	0.00	1784	0.00	-	-	1784	0.00	1784	0.00	1784	0.00
LA32	30×10	1850	1850	0.00	-	-	1850	0.00	1850	0.00	1850	0.00	-	-	1868	0.97	1851	0.05	1850	0.00
LA33	30×10	1719	1719	0.00	-	-	1719	0.00	1719	0.00	1719	0.00	-	-	-	-	1719	0.00	1719	0.00
LA34	30×10	1721	1721	0.00	-	-	1725	0.23	1788	3.89	1721	0.00	-	-	-	-	1749	1.62	1721	0.00
LA35	30×10	1888	1888	0.00	-	-	1888	0.00	1947	3.125	1888	0.00	-	-	1901	0.68	1888	0.00	1888	0.00
LA36	15×15	1268	1295	2.12	1304	2.83	1308	3.15	1388	9.46	1311	3.39	-	-	-	-	1334	5.20	-	-
LA37	15×15	1397	1415	1.28	-	-	1489	6.58	1486	6.37	-	-	-	-	-	-	1467	5.01	-	-
LA38	15×15	1196	1246	4.18	-	-	1275	6.60	1339	11.95	-	-	-	-	1258	5.18	1278	6.85	-	-
LA39	15×15	1233	1258	2.02	-	-	1290	4.62	1334	8.19	-	-	-	-	-	-	1296	5.10	-	-
LA40	15×15	1222	1243	1.71	1252	2.45	1252	2.45	1347	10.22	-	-	-	-	-	-	1284	5.07	-	-
MErr				**0.61**		**0.59**		**1.50**		**3.52**		**1.27**		**0.38**		**0.77**		**1.99**		**0.80**
FT06	6×6	55	55	0.00	55	0.00	55	0.00	-	-	55	0.00	55	0.00	55	0.00	55	0.00	55	0.00
FT10	10×10	930	930	0.00	936	0.64	935	0.53	-	-	940	1.07	951	2.25	937	0.75	960	3.22	930	0.00
FT20	20×5	1165	1165	0.00	1165	0.00	1180	1.28	-	-	1178	1.11	1178	1.11	1182	1.45	1192	2.31	1165	0.00
MErr				**0.00**		**0.21**		**0.60**		-		**0.73**		**1.12**		**0.73**		**1.84**		**0.00**
ORB01	10×10	1059	1068	0.84	-	-	1084	2.36	-	-	-	-	-	-	-	-	1099	3.77	1092	3.11
ORB02	10×10	888	889	0.11	-	-	890	0.22	-	-	-	-	-	-	-	-	906	2.02	894	0.67
ORB03	10×10	1005	1023	1.79	-	-	1037	3.18	-	-	-	-	-	-	-	-	1056	5.07	1029	2.38
ORB04	10×10	1005	1005	0.00	-	-	1028	2.28	-	-	-	-	-	-	-	-	1032	2.68	1016	1.09
ORB05	10×10	887	889	0.22	-	-	894	0.78	-	-	-	-	-	-	-	-	909	2.48	901	1.57
ORB06	10×10	1010	1019	0.89	-	-	1035	2.47	-	-	-	-	-	-	-	-	1038	2.77	1028	1.78
ORB07	10×10	397	397	0.00	-	-	404	1.76	-	-	-	-	-	-	-	-	411	3.52	405	2.01
ORB08	10×10	899	907	0.88	-	-	937	4.22	-	-	-	-	-	-	-	-	917	2.00	914	1.66
ORB09	10×10	934	940	0.64	-	-	943	0.96	-	-	-	-	-	-	-	-	-	-	943	0.96
ORB10	10×10	944	944	0.00	-	-	967	2.43	-	-	-	-	-	-	-	-	-	-	-	-
MErr				**0.54**		-		**2.07**		-		-		-		-		**3.04**		**1.69**
ABZ05	10×10	1234	1234	0.00	-	-	1238	0.32	-	-	-	-	-	-	-	-	1241	0.56	-	-
ABZ06	10×10	943	943	0.00	-	-	947	0.42	-	-	-	-	-	-	-	-	964	2.22	-	-
ABZ07	20×15	656	695	5.94	-	-	-	-	-	-	-	-	-	-	-	-	719	9.60	-	-
ABZ08	20×15	665	713	10.03	-	-	-	-	-	-	-	-	-	-	-	-	738	13.88	-	-
ABZ09	20×15	679	721	6.34	-	-	-	-	-	-	-	-	-	-	-	-	742	9.43	-	-
MErr				**4.46**		-		**0.37**		-		-		-		-		**7.14**		-

**Table 6:** Set up for case study III. Where $c_{\mathrm{best}}$ is the best individual in the population and $c_{\mathrm{best},1}$ and $c_{\mathrm{best},2}$ are the two best individuals in the population who are different.

	GA	GSA	LSGA	aLSGA	mXLSGA
Population size	100	100	100	100	100
Generations	100	100	100	100	100
Crossover rate	0.95	0.95	0.95	0.95	0.95
Mutation rate	0.05	0.05	1	1	1
$R_{c}$	1	10	1	1	10
$R_{m}$	1	140	N·M	N·M	N·M
$F_{\times }$	$\left\{\mathrm{PMX}\right\}$	$\left\{\mathrm{PMX}\right\}$	$\left\{\mathrm{PMX}\right\}$	$\left\{\mathrm{PMX}\right\}$	$\left\{\mathrm{OX}2,\mathrm{PMX}\right\}$
$F_{\mathrm{mut}}$	$\left\{f_{\mathrm{swap}}\right\}$	$\left\{f_{\mathrm{swap}}\right\}$	$\left\{f_{\mathrm{swap}}\right\}$	$\left\{f_{\mathrm{swap}},f_{\mathrm{inverse}},f_{\mathrm{insert}}\right\}$	$\left\{f_{\mathrm{swap}},f_{\mathrm{inverse}},f_{\mathrm{insert}}\right\}$
$F_{\mathrm{pert}}$				$\left\{f_{\mathrm{swap}}\right\}$	$\left\{f_{\mathrm{swap}},f_{\mathrm{inverse}},f_{\mathrm{insert}}\right\}$
$\epsilon_{\mathrm{LS}}$	0	0	0.5	1	0.95
$P_{\mathrm{massive}}$				$\left\{c_{\mathrm{best}}\right\}$	$\left\{c_{\mathrm{best},1},c_{\mathrm{best},2}\right\}$

**Table 7:** Genetic Algorithm (GA)-like methods statistics. The symbol “-” means that the method was not able to reach the optimum solution.

Instance	Method	Best	Worst	Average	SD	Number Opt	Number It	AT (s)
	GA	55	57	55.45	0.85	27	52	2.4
	GSA	55	55	55	0	35	8	39.78
FT 06	LSGA	55	59	57.68	1.43	6	27	7.95
	aLSGA	55	55	55	0	35	11	11.51
	mXLSGA	55	55	55	0	35	5	22.11
	GA	666	712	679.02	9.98	6	76	2.65
	GSA	666	715	677.8	13.61	13	10	51.79
LA 01	LSGA	666	726	697	16.65	1	61	12.15
	aLSGA	666	666	666	0	35	11	20.07
	mXLSGA	666	666	666	0	35	5	38.06
	GA	926	938	927.4	2.87	24	79	3.34
	GSA	926	935	926.31	1.54	33	7	67.31
LA 06	LSGA	926	970	935.8	13.15	17	55	19.87
	aLSGA	926	926	926	0	35	3	38.07
	mXLSGA	926	926	926	0	35	2	71.98
	GA	1222	1256	1235.97	10.81	5	58	3.97
	GSA	1222	1276	1232.14	14.94	20	19	81.59
LA 11	LSGA	1222	1299	1251.6	19.62	2	32	31.33
	aLSGA	1222	1222	1222	0	35	5	60.82
	mXLSGA	1222	1222	1222	0	35	3	116.57
	GA	982	1100	1045.6	26.40	0	-	2.89
	GSA	994	1110	1046.77	26.37	0	-	55.31
LA 16	LSGA	1016	1148	1084.25	32.27	0	-	20.62
	aLSGA	959	985	980.51	4.48	0	-	38.75
	mXLSGA	945	982	972.25	13.30	2	96	66.25
	GA	1189	1336	1271.71	34.44	0	-	3.78
	GSA	1148	1347	1214.08	43.85	0	-	73.19
LA 23	LSGA	1214	1419	1295.34	43.70	0	-	38.39
	aLSGA	1035	1115	1078	16.34	0	-	75.62
	mXLSGA	1032	1093	1060.45	17.96	1	51	123.64
	GA	1525	1699	1619.51	39.50	0	-	4.44
	GSA	1433	1586	1512.22	36.47	0	-	91.67
LA 26	LSGA	1517	1665	1597.94	36.67	0	-	60.84
	aLSGA	1302	1384	1343.28	19.69	0	-	124.93
	mXLSGA	1218	1371	1300.85	41.88	6	85	203.95
	GA	2120	2326	2223.14	48.45	0	-	6.23
	GSA	1943	2142	2050.17	63.09	0	-	130.75
LA 31	LSGA	2005	2336	2177	66.29	0	-	123.23
	aLSGA	1808	1897	1843.51	21.17	0	-	258.53
	mXLSGA	1784	1845	1807.71	19.20	5	80	424.56

**Table 8:** Result of 35 independent executions of each method considering as stopping criterion the execution time. In this case, in each execution, all techniques are performed for, at least, the same amount of time as the technique that takes the longest time in the considered instance.

Instance	Method	Best	Worst	Average	SD	Number opt	AT (s)
	GA	55	55	55	0	35	39.78
	GSA	55	55	55	0	35	39.78
FT 06	LSGA	55	55	55	0	35	39.78
	aLSGA	55	55	55	0	35	39.78
	mXLSGA	55	55	55	0	35	39.78
	GA	666	688	668.80	6.13	24	51.79
	GSA	666	715	677.8	13.61	13	51.79
LA 01	LSGA	666	731	692.11	13.66	2	51.79
	aLSGA	666	666	666	0	35	51.79
	mXLSGA	666	666	666	0	35	51.79
	GA	926	926	926	0	35	71.98
	GSA	926	937	926.37	1.88	33	71.98
LA 06	LSGA	926	926	926	0	35	71.98
	aLSGA	926	926	926	0	35	71.98
	mXLSGA	926	926	926	0	35	71.98
	GA	1222	1222	1222	0	35	116.57
	GSA	1222	1267	1226.54	9.49	25	116.57
LA 11	LSGA	1222	1222	1222	0	35	116.57
	aLSGA	1222	1222	1222	0	35	116.57
	mXLSGA	1222	1222	1222	0	35	116.57
	GA	982	1035	997.74	18.36	0	66.25
	GSA	993	1103	1044.51	28.94	0	66.25
LA 16	LSGA	1003	1138	1081.48	31.66	0	66.25
	aLSGA	959	985	979.2	3.57	0	66.25
	mXLSGA	945	982	972.25	13.30	2	66.25
	GA	1042	1180	1104.68	27.51	0	123.64
	GSA	1133	1262	1201.65	31.33	0	123.64
LA 23	LSGA	1224	1353	1276.02	35.10	0	123.64
	aLSGA	1037	1106	1069.97	19.53	0	123.64
	mXLSGA	1032	1093	1060.45	17.96	1	123.64
	GA	1309	1449	1382.97	33.59	0	203.95
	GSA	1421	1599	1515.91	49.36	0	203.95
LA 26	LSGA	1496	1690	1591.20	48.03	0	203.95
	aLSGA	1285	1374	1332.11	22.97	0	203.95
	mXLSGA	1218	1371	1300.85	41.88	6	203.95
	GA	1810	2022	1886.34	48.18	0	424.56
	GSA	1974	2176	2056.80	51.09	0	424.56
LA 31	LSGA	2005	2261	2155.54	51.48	0	424.56
	aLSGA	1808	1866	1821.80	20.90	0	424.56
	mXLSGA	1784	1845	1807.71	19.20	5	424.56
